# Development of a Virtual Reality Coping Skills Game to Prevent Post-Hospitalization Smoking Relapse in Tobacco Dependent Cancer Patients

**Published:** 2009-08

**Authors:** Paul Krebs, Jack Burkhalter, Shireen Lewis, Tinesha Hendrickson, Ophelia Chiu, Paul Fearn, Wendy Perchick, Jamie Ostroff, Memorial Sloan-Kettering Cancer Center

**Keywords:** smoking cessation, health behavior, virtual reality

## Abstract

Many hospitalized smokers return to smoking after hospital discharge even though continued smoking can compromise treatment effectiveness, reduce survival, increase risk of disease recurrence, and impair quality of life. After leaving a smoke-free hospital, patients encounter smoking cues at home, such as family members who smoke or emotional triggers such as stress, which can elicit powerful urges to smoke and lead to smoking relapse. Enabling smokers to experience such urges in a controlled setting while providing the ability to practice coping skills may be a useful strategy for building quitting self-efficacy. We are developing a virtual reality coping skills (VRCS) game to help hospitalized smokers practice coping strategies to manage these triggers in preparation for returning home after hospitalization. Our multidisciplinary team developed a prototype VRCS game using Second Life, a platform that allowed rapid construction of a virtual reality environment. The prototype contains virtual home spaces (e.g., living room, kitchen) populated with common triggers to smoke and a “toolkit” with scripted actions that enable the avatar to rehearse various coping strategies. Since eliciting and managing urges to smoke is essential to the game’s utility as an intervention, we assessed the ability of the prototype virtual environment to engage former smokers in these scenarios. We recruited eight former smokers with a recent history of hospitalization and guided each through a VRCS scenario during which we asked the patient to evaluate the strength of smoking urges and usefulness of coping strategies. Initial data indicate that patients report high urges to smoke (mean = 8.8 on a 10 point scale) when their avatar confronted virtual triggers such as drinking coffee. Patients rated virtual practice of coping strategies, such as drinking water or watching TV, as very helpful (mean = 8.4 on a 10 point scale) in reducing these urges. With further development, this VRCS game may have potential to provide low-cost, effective behavioral rehearsal to prevent relapse to smoking in hospitalized patients.

Of the 39 million Americans hospitalized in 2005, 8 million are estimated to have been smokers, using a smoking prevalence of 20.9% (Centers for Disease Control, 2007). While most smokers are able to abstain from smoking during inpatient stays within the smoke-free hospital environment, resumption of smoking following hospital discharge from the smoke-free hospital is common (Emmons & Goldstein, 1992; France, Glasgow, & Marcus, 2001; MacKenzie, Pereira, & Mehler, 2004; Wolfenden, 2003). Typical triggers for smoking relapses are distress (both psychological and physical), other household smokers, and environmental smoking cues. Higher self-efficacy (confidence) for managing smoking urges is a consistent predictor of quitting success (Ockene, et al., 2000), while low self-efficacy for coping with smoking urges can leave a smoker particularly vulnerable to these triggers.

High rates of return to smoking have stimulated extensive research effort and theory development directed toward relapse prevention (Marlatt & Gordon, 1985; USDHHS, 2008), but outcomes for relapse prevention interventions have been mixed. A recent systematic review of smoking relapse prevention studies found no benefit from trials typically using low-intensity interventions, such as brief face-to-face encounters, written materials, mailings, and telephone contact (Lancaster, Hajek, Stead, West, & Jarvis, 2006). More intensive interventions involving hospital-based and post-discharge counseling, however, do show promise (Rigotti, Munafo, & Stead, 2008). Given the high relapse rates and the health risks of continued smoking, novel approaches to relapse prevention are needed.

The standard behavioral treatment for smoking urges has been behavioral rehearsal, which entails the identification, modeling, and role-playing of diverse cognitive and behavioral coping strategies. This treatment promotes skill acquisition and mastery, bolsters confidence in coping with smoking cues, and reduces relapse to smoking (Irvin, Bowers, Dunn, & Wang, 1999). By enabling patients to interact with realistic environments, virtual reality environments are uniquely suited to engaging recent ex-smokers in skill-building activities for managing urges to smoke.

Through exposure to conditioned cues to smoke, (e.g., socializing with friends who smoke), smokers may be able to virtually practice coping skills and build crucial self-efficacy skills for resisting smoking urges. In initial trials, virtually-presented cues have been shown to elicit more cravings than static photographs presented during traditional therapies (Baumann & Sayette, 2006; Bordnick, et al., 2004; Carter, Bordnick, Traylor, Day, & Paris, 2008; Kuntze, et al., 2001; Lee, et al., 2003). In contrast to “laboratory”-focused virtual craving studies, our project has focused on direct clinical application using a virtual-reality-based intervention to help smokers prevent relapse to tobacco use. Only one study (Nemire, 1999) has attempted a virtual reality *intervention* related to smoking, creating a virtual park where “avatars” (computer-generated people) offered cigarettes to adolescents. Participants could interact with the avatars and rehearse strategies for refusing cigarettes. Those using the virtual game acquired superior refusal skills compared to those receiving traditional life skills training. Despite the promise such interventions hold, no study has yet examined the efficacy of using a virtual reality intervention to promote smoking cessation and prevent smoking relapse among adult smokers.

The overall aim of our current project is to develop a prototype virtual reality coping skills (VRCS) simulation game to help hospitalized smokers increase their skill and confidence in managing post-hospital discharge smoking cues. We chose to focus on the development of a Virtual Reality Coping Skills (VRCS) because a VRCS game: a) can be individualized to address a patient’s specific smoking triggers; b) can create realistic simulations that provide behavioral rehearsal opportunities that are either not possible or unethical in real-world treatment settings; c) can be readily disseminated to a broad audience of hospitalized smokers with minimal resource burden; and d) may be cost-effective as the initial outlay of costs for an effective game can be recouped with wide access and efficiency through reduced therapist/cessation counselor time.

Prior to creating an intervention game, however, we wanted to gather preliminary “proof-of-concept” and usability data to inform eventual intervention development. We hypothesize that a virtual reality game used by hospitalized smokers can simulate high risk triggers for smoking relapse and help smokers master effective coping strategies to manage these triggers in preparation for discharge to home environments saturated with smoking cues. This preliminary stage is vital to ensuring a final product that will have the most utility in achieving our goal of moving the proposed project into the next phase of research in which we will examine the utility of the VRCS with actual inpatients at risk for smoking relapse after hospital discharge.

## Method

### Virtual World Development

A multidisciplinary team of investigators from the Behavioral Sciences Service (Krebs, Burkhalter, Ostroff) and the Strategic Planning and Innovation Department (Perchick, Fearn, Lewis, Chiu, Hendrickson) met over a 6-month period to plan a VRCS game using recommended game development methods (deFreitas, 2006; Garris, Ahlers, & Driskell, 2002). The process of creating virtual elements has been a collaborative process that relies on instructional content input from behavioral scientists, input from technology and gaming experts, and feedback from former smokers representing end users.

To begin development of the VRCS game, the MSKCC Strategic Planning and Innovation team leased a private “sim” in Linden Lab’s Second Life (SL) (Linden Lab, 2009), which is a real-time virtual environment where users have the ability to create customized “avatar” characters to represent themselves. In SL these avatars have the ability to explore virtual islands, or “sims,” ranging from recreational places to islands with educational purpose. Several organizations have begun to use SL as a venue for support groups, social networking, and to provide didactic education.

While we ultimately envision our smoking cessation program to operate more like a self-contained “game” with program-generated prompts and feedback, we employed SL (which is not a game but a virtual “world”) (New Media Consortium and the Educase Learning Initiative, 2007) because it is an ideal platform for rapidly creating virtual environments. Our aim at this developmental phase was to create a realistic environment to test the feasibility and acceptability of presenting smoking cues and coping strategies to hospitalized smokers in a virtual world. With content consultation from behavioral scientists knowledgeable in tobacco cessation treatment, the Innovation team worked with a programmer to build the virtual spaces, create custom avatars and props, and develop scripts for particular movements and interactions. Consistent with cognitive-behavioral counseling techniques used in smoking cessation treatment, we created a virtual environment with the following features:

- Virtual spaces (living room, kitchen, office, and sidewalk in front of office building) populated with challenges for a patient trying to resist smoking urges, such as sitting at a computer or watching television.
- Illness-specific contexts, such as experiencing distress or pain (via a scripted action in which the avatar engages in anxious behavior such as holding a hand to the forehead).
- “Guest avatars” that smoke and elicit social smoking cues for the patient to manage.
- A “coping toolbox” that when touched presents techniques the patient can use to cope with urges to smoke (e.g., exercising on treadmill, having a drink of water or a snack, engaging in deep breathing).

### Prototype Testing

#### Sample

We recruited participants via telephone using the patient database of an urban hospital-based smoking cessation clinic in the northeastern United States. Participants had recently quit smoking, had been hospitalized within the past year for a cancer-related illness, and had adequate visual-manual dexterity to participate. Our institutional review board classified this developmental work as quality improvement and therefore did not require approval as human subjects research.

#### Testing protocol

Since the virtual environment’s ability to elicit urges to smoke and to allow patients to practice coping strategies is essential to the intervention’s central premise, each patient was guided through two VRCS scenarios populated with common environmental smoking triggers and cues (such as drinking coffee). Patients could also create their own tempting situations and coping strategies using elements present in the environment and SL interface (such as taking a walk outside). As our primary interest was eliciting and managing temptations during this evaluation phase, a project staff member (Krebs, Lewis) operated the user interface to control for patient familiarity with the operation of SL. For each scenario we asked participants how tempted they felt to smoke using a scale of 1–10, with 10 designated as the greatest possible temptation. After each tempting scenario we directed each participant to the coping toolbox where he/she selected a coping strategy, observed their avatar use the strategy, and then rated its ability in helping them cope with the urge to smoke using a 1–10 scale, with 10 representing the most helpful in reducing the urge. After two guided iterations of temptation and coping strategy scenarios, we allowed patients a few minutes to explore the environment using the computer on their own. We then asked a series of overall evaluative questions encompassing domains of realism and usability. Testing was conducted at the hospital’s Smoking Cessation Clinic and each session was video-taped, viewed, and coded by both the lead author and SL.

## Results

### Participants

Eight participants (7 female) completed an evaluation of the VRSC game. Their mean age was 48 (range 28 to 73). Seven identified as White and one as Black. The average length of program evaluation session was 40 minutes.

### Temptations

Participants chose the following scenarios to elicit temptation to smoke: typing at a computer (2), sorting papers at a desk (1), drinking coffee (5), watching TV (3), sitting with a smoker (3), taking dog for a walk (1), and cooking (1). The average temptation score for these situations was 8.8 (range = 5 to 10). Suggestions for additional tempting situations were: having an alcoholic drink (5), talking on the phone (3), socializing with others (4), sitting in a car (1), lying in bed (1), and finishing a meal (1).

### Coping Strategies

Strategies that participants chose to cope with urges to smoke were: looking out the window (1), watching TV (1), using nicotine inhaler (1), leaving the situation (1), exercising (2), “asking” a smoker to not smoke around them (1), washing dishes (1), thinking about effects of smoking on others (1), and having a glass of water (1). Participants’ average rating score for the helpfulness of situations in reducing their urge to smoke was 8.4 (range = 5 to 10). Additional coping strategies participants suggested adding were: getting rid of ashtrays (2), practicing cognitive techniques such as thought-stopping or thinking of cons of smoking (3), cooking (1), listening to music (1), taking a nap (1), eating a snack (3), getting a pet (1), being able to ask the smoking avatar to put out the cigarette (3), using nicotine replacement (1), chewing gum (1), reading (2), shopping (1), and completing a puzzle (1).

### Design and Usability

Upon completion of the scenarios, we asked a series of questions to evaluate the design and usability of the game. Response categories, mean ratings, and selected comments are shown in Table 1.

Most (n = 6) participants noted that they would prefer to move the avatar using the mouse, which was not possible in the prototype given that the interface was governed by Second Life. Additional suggestions were that we arrange the furniture to make the room easier to walk though and add more contrast between colors. One patient noted that the unrealistic eating and drinking movements made it difficult for her to engage as the character. To make the game more interesting, a few patients suggested greater interactivity such as allowing patients to load the tool box with their own coping strategies, earning points and rewards, having more options to interact with objects (open drawers, pick up books), and the ability to add sounds such as telephones ringing or the sound of typing.

In terms of its current status and potential to help patients prepare to quit smoking, participants noted, for example “I think it would help people because when they get home they forget about challenges, like seeing the bed or somebody on the couch as automatic cues;” “If the computer interacted it would be fairly helpful;” and “I like the idea of having another modality to rehearse, practice, and prepare.”

## Discussion

The qualitative and quantitative feedback that we gathered from former smokers indicated three primary conclusions regarding the potential for a virtual reality smoking cessation intervention. First, virtually presented smoking cues can elicit moderate to high levels of temptation to smoke. Second, virtually presented coping strategies can reduce temptation to smoke, and third having an engaging design and high usability are essential for creating a program that smokers would find beneficial.

The finding that virtual cues elicit temptation to smoke is consistent with the limited number of other studies showing that nicotine craving can be elicited in virtual environments (Bordnick, et al., 2004; Carter, et al., 2008; Lee, et al., 2003). These studies, however, employed head-mounted displays which are costly and not feasible for most treatment environments or for home use. Most similar to our study was one conducted by Baumann & Sayette (2006) that used a standard computer monitor and found that a free exploration of an outdoor environment containing smoking cues elicited stronger urges to smoke than exploration of an environment without such cues.

While research has largely established the ability of virtual cues to generate nicotine craving, our pilot project is the only study we know of that assessed real-time reactions to virtual coping scenarios. For instance, the virtual reality program Nemire (1999) created was directed at nonsmokers, allowing them to practice only refusal strategies. Our preliminary finding that enactment of coping skills in the virtual environment may alleviate urges to smoke provides initial “proof of concept” support for the development of the proposed virtual reality-based cessation intervention.

The qualitative data, in particular, provided useful direction for creating a virtual-reality intervention that patients would use. There was a clear consensus that patients would prefer a program that operated more like an interactive computer game with prompts, feedback, ability levels, and a reward/point system. Our trial relied on the therapist to guide participants to cues and strategies, and patients noted that exploring a virtual world without prompts or interaction from the computer would not be helpful. Environments such as one created by Baumann & Sayette (2006), which simply enable participants to walk through situations containing cues, do not necessarily produce opportunities to practice coping strategies. Participants also emphasized the importance of the ability to personalize their avatars, as most noted they did not identify with the “neutral” avatar we created. They also stated that they would not engage in a program that proved frustrating to use. Therefore labels and text need to be clear and large, avatars should be intuitively movable via a mouse, and the program should start with a brief training module. There were no apparent age differences in usability, diminishing our initial concerns regarding the acceptability of this program for hospitalized smokers, many of whom are older adults.

The feedback we gathered largely supported the importance of “situated learning” (Brown, Collins, & Duguid, 1989), such that problem solving is best carried out in conjunction with the environment and that concepts are best learned in realistic situations. While traditional smoking cessation interventions often provide smokers with a list of “coping strategies,” such as distraction and stimulus avoidance, these directives are abstract and not as readily acquired outside a specific environmental learning context. The importance of situated learning is supported by a number of comments we received such as, “Instead of talking about what you would do, now you can actually go and do it” and “You remember things as you are doing them [so it could] definitely help you plan for cues.” Our trials also highlighted a point made by Schneider (1996) that virtual environments must be adaptable and should contain objects that can be manipulated in a natural manner. Indeed, while we prepared the environment with common smoking triggers and strategies, participants instead used the environment in unexpected ways, such as having their avatar stand at the sink as if washing dishes or walking outside to cope with smoking cues. Participants also noted, for example, that they would be more engaged if they could touch and move objects such as opening drawers, throwing away ashtrays, preparing food, etc.

### Limitations

Our data was limited due to the nature of the volunteer, convenience sample. Our sample was primarily (7/8) female, white (7/8), and composed of those who quit smoking within the past year. It should also be noted that while much of the literature regarding virtual reality interventions focuses on Web 2.0 social-networking capabilities, our prototype does not make use of this capability. The goal of this intervention is to develop tobacco cessation skills rather than to provide nonspecific social support, although this latter element could be incorporated in future intervention development phases.

## Conclusions

While a Virtual-Reality Coping-Skills game may not be suitable for every hospitalized smoker, its development holds promise as an additional treatment modality. Tobacco cessation is characterized by chronic relapse, necessitating engaging methods to tailor treatment to a person’s unique triggers and situation. As noted in the Horizon Report and elsewhere (Gorini, Gaggioli, Vigna, & Riva, 2008; New Media Consortium and the Educase Learning Initiative, 2007), integration of real-time data capture devices, such as phones, with the virtual environment offers the possibility of increasing the immersive nature and feedback quality of virtual interventions. With further development as an interactive game, the virtual environment we have created has the potential to provide low-cost, effective behavioral rehearsal to prevent relapse to smoking in hospitalized patients. The process we undertook of collaborative, systematic development and end-use feedback has enabled our team to confidently pursue creation of a virtual-reality coping skills game and to implement a research trial that would test its efficacy in reducing relapse to smoking.

## Figures and Tables

**Figure 1:**
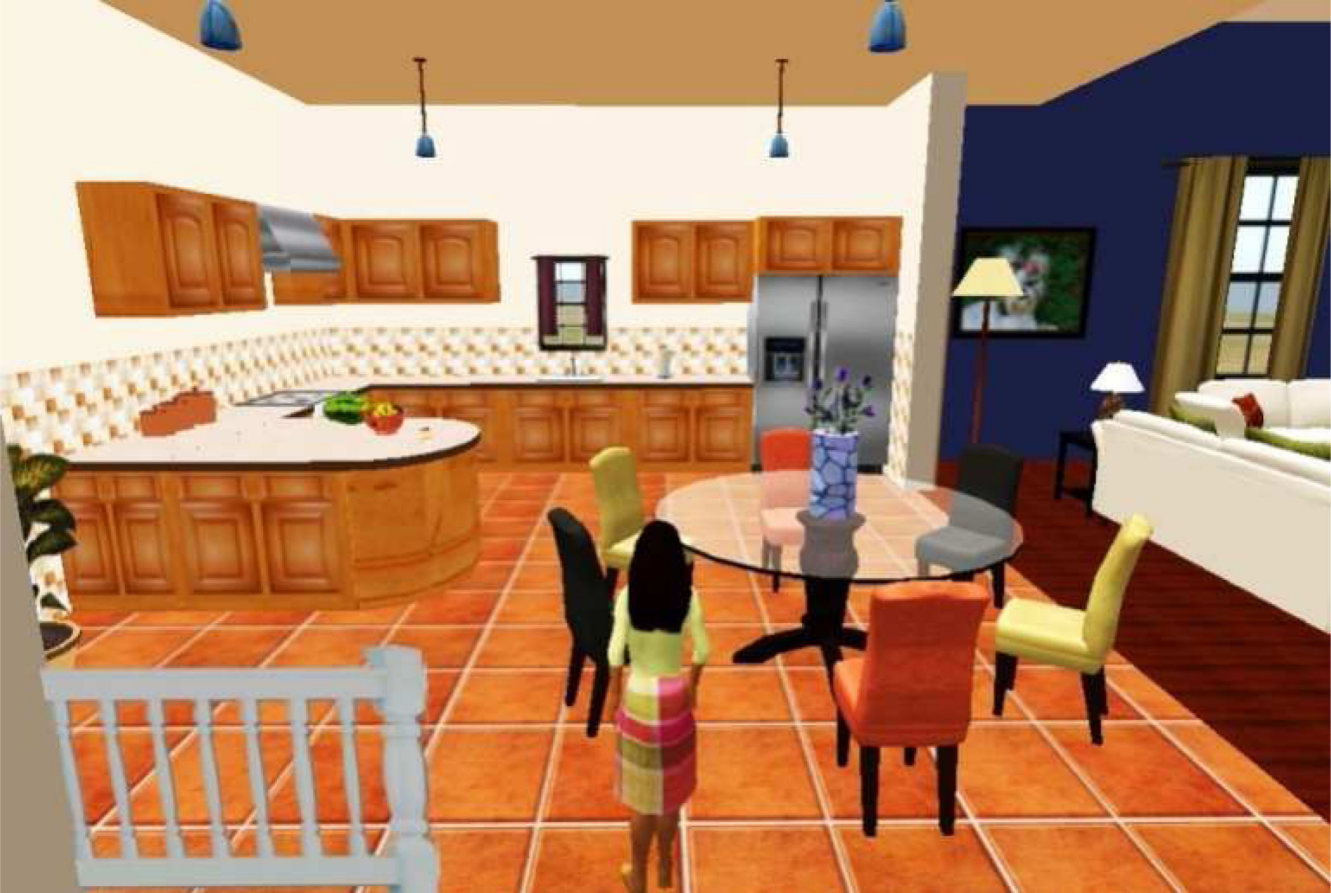
Female avatar in virtual dining and living room area created for the project

**Table 1:** Response categories, mean ratings, and selected comments

Outcome	Mean (SD) (1–10 scale)	Representative comments
Stimulating	5.6 (2.2)	“People would be more interested if they had options to touch things”
		“I like games more quick-moving”
		“Even though there’s not enough activities going on, the architecture of it is pretty good”
		“For me it was fun and interesting but I don’t relate to it because this is a big fancy house and I live in a studio apartment”
Easy to Use	5.4 (1.6)	“I didn’t navigate too well, but with a little practice it wasn’t too difficult”
		“Would be easier if you could just drag her [avatar] along”
		“Frustrating because navigating her [avatar] around is difficult”
		“Really good program but frustrating to manipulate”
Helpful	6.5 (2.2)	“Not sure if it would help me unless it did something like a game, like ability to earn points”
		“The more interactive it is the more helpful it would be”
Ability to engage as character	4.4 (3.1)	“I could certainly be her”
		“I didn’t engage as the character at all. I don’t have gray hair”
		“Probably if you could change the clothes”
		“The character doesn’t look like me—that’s a big part of it”
		“Need to give more layout options for the house”
		“Not enough activities to do”
Clarity of labels	8.2 (1.5)	“Not confusing at all”
		“Need to be bigger”

## References

[R1] Baumann SB, Sayette MA (2006). Smoking Cues in a Virtual World Provoke Craving in Cigarette Smokers. Psychology of Addictive Behaviors.

[R2] Bordnick PS, Graap KM, Copp H, Brooks J, Ferrer M, Logue B (2004). Utilizing Virtual Reality to Standardize Nicotine Craving Research: A Pilot Study. Addictive Behaviors.

[R3] Brown JS, Collins A, Duguid P (1989). Situated Cognition and the Culture of Learning. Educational Researcher.

[R4] Carter BL, Bordnick P, Traylor A, Day SX, Paris M (2008). Location and Longing: The Nicotine Craving Experience in Virtual Reality. Drug and Alcohol Dependence.

[R5] Centers for Disease Control (2007). Cigarette Smoking Among Adults - United States, 2006. MMWR.

[R6] De Freitas S (2006). Learning in Immersive Worlds: A Review of Game-Based Learning.

[R7] Emmons KM, Goldstein MG (1992). Smokers Who Are Hospitalized: A Window of Opportunity for Cessation Interventions. Preventive Medicine.

[R8] France EK, Glasgow RE, Marcus AC (2001). Smoking Cessation Interventions among Hospitalized Patients: What Have We Learned?. Preventive Medicine.

[R9] Garris R, Ahlers R, Driskell JE (2002). Games, Motivation, and Learning: A Research and Practice Model. Simulation & Gaming.

[R10] Gorini A, Gaggioli A, Vigna C, Riva G (2008). A Second Life for eHealth: Prospects for the Use of 3-D Virtual Worlds in Clinical Psychology. Journal of Medical Internet Research.

[R11] Irvin JE, Bowers CA, Dunn ME, Wang MC (1999). Efficacy of relapse prevention: A meta-analytic review. Journal of Consulting and Clinical Psychology.

[R12] Kuntze MMF, Stoermer RR, Mager RR, Roessler AA, Mueller-Spahn FF, Bullinger AAH (2001). Immersive Virtual Environments in Cue Exposure. Cyberpsychology & Behavior.

[R13] Lancaster T, Hajek P, Stead LF, West R, Jarvis MJ (2006). Prevention of Relapse after Quitting Smoking: A Systematic Review of Trials. Archives of Internal Medicine.

[R14] Lee JH, Ku J, Kim K, Kim B, Kim IY, Yang BH (2003). Experimental Application of Virtual Reality for Nicotine Craving through Cue Exposure. Cyberpsychology and Behavior.

[R15] Linden Lab (2009). Second Life.

[R16] MacKenzie TD, Pereira RI, Mehler PS (2004). Smoking Abstinence after Hospitalization: Predictors of Success. Preventive Medicine.

[R17] Marlatt GA, Gordon JR (1985). Relaspe Prevention: Maintenance Strategies in the Treatment of Addictive Behaviors.

[R18] Nemire KK (1999). Preventing Teen Smoking with Virtual Reality. Cyberpsychology & Behavior.

[R19] New Media Consortium and the Educase Learning Initiative (2007). The Horizon Report.

[R20] Ockene JK, Emmons KM, Mermelstein RJ, Perkins KA, Bonollo DS, Voorhees CC (2000). Relapse and maintenance issues for smoking cessation. Health Psychology.

[R21] Rigotti NA, Munafo MR, Stead LF (2008). Smoking Cessation Interventions for Hospitalized Smokers: A Systematic Review. Archives of Internal Medicine.

[R22] Schneider DK (1996). Virtual Environments for Education, Research and Life.

[R23] USDHHS (2008). Treating tobacco use and dependence: Clinical practice guideline 2008 update.

[R24] Wolfenden L (2003). Smoking Cessation Interventions for In-patients: A Selective Review with Recommendations for Hospital-based Health Professionals. Drug and Alcohol Review.

